# *VKORC1* Pharmacogenetics and Pharmacoproteomics in Patients on Warfarin Anticoagulant Therapy: Transthyretin Precursor as a Potential Biomarker

**DOI:** 10.1371/journal.pone.0015064

**Published:** 2010-12-13

**Authors:** Ramasamy Saminathan, Jing Bai, Laleh Sadrolodabaee, Govindasamy Muralidharan Karthik, Onkar Singh, Koilan Subramaniyan, Chi Bun Ching, Wei Ning Chen, Balram Chowbay

**Affiliations:** 1 Laboratory of Clinical Pharmacology, Division of Medical Sciences, Humphrey Oei Institute of Cancer Research, National Cancer Centre, Singapore, Singapore; 2 School of Chemical and Biomedical Engineering, College of Engineering, Nanyang Technological University, Singapore, Singapore; University of California Los Angeles and Cedars-Sinai Medical Center, United States of America

## Abstract

**Background:**

Recognizing specific protein changes in response to drug administration in humans has the potential for the development of personalized medicine. Such changes can be identified by pharmacoproteomics approach based on proteomic technologies. It can also be helpful in matching a particular target-based therapy to a particular marker in a subgroup of patients, in addition to the profile of genetic polymorphism. Warfarin is a commonly prescribed oral anticoagulant in patients with prosthetic valve disease, venous thromboembolism and stroke.

**Methods and Finding:**

We used a combined pharmacogenetics and iTRAQ-coupled LC-MS/MS pharmacoproteomics approach to analyze plasma protein profiles of 53 patients, and identified significantly upregulated level of transthyretin precursor in patients receiving low dose of warfarin but not in those on high dose of warfarin. In addition, real-time RT-PCR, western blotting, human IL-6 ELISA assay were done for the results validation.

**Conclusion:**

This combined pharmacogenomics and pharmacoproteomics approach may be applied for other target-based therapies, in matching a particular marker in a subgroup of patients, in addition to the profile of genetic polymorphism.

## Introduction

Warfarin is an oral anticoagulant commonly employed in the treatment and prevention of thromboembolic events such as myocardial infarction, atrial fibrillation and deep vein thrombosis [1], [2]. However, large inter- and intra-individual variabilities in treatment responses coupled with a narrow therapeutic range have made the clinical optimization of warfarin doses difficult. The dose requirements for warfarin have been shown to be influenced by various factors including age, weight, ethnicity, vitamin-K enriched diet, drug interactions and genetics of individuals [3], [4], [5], [6], [7], [8]. Current clinical practice utilizes the international normalization ratio (INR) to optimize the dose of warfarin in individual patients which has performed far from ideal. The pharmacogenetics of warfarin has been the focus of recent research to elucidate the factors which can influence the dose of warfarin and identify the biomarkers which predict the optimal warfarin doses [9], [10], [11].

Warfarin is an orally administered coumarin derivative which is rapidly absorbed into the systemic circulation with bioavailability of 100%. Up to 99% of the circulating drug is bound to plasma albumin and alpha-1-acid glycoprotein. Warfarin is present as a racemic mixture of S- and R- enantiomers with S-warfarin being 3 to 5 times more active than the R-enantiomer [12], [13]. Besides the activity, the metabolic profiles of the 2 enantiomers have also been found to differ. The metabolism of S-warfarin to its inactive metabolite, 7-hydroxywarfarin, is predominantly catalyzed by *CYP2C9* (Cytochrome P450 2C9) with minor contributions from *CYP2C8* and *CYP2C19*. In contrast, R-warfarin is mainly metabolized by *CYP1A2* and *CYP3A4* to form the inactive metabolites, 8-hydroxywarfain and 10-hydroxywarfain, respectively [12]. Other enzymes which play minor roles in this metabolic pathway include *CYP1A1, CYP2C8, CYP2C9* and *CYP3A5*. The impact of *CYP2C9* polymorphic variants on the pharmacokinetics and pharmacodynamics of warfarin has been extensively studied in patients from different ethnic backgrounds [14], [15], [16], [17]. In particular, CYP2C9*2 and *3 polymorphisms have been associated with greater risk of bleeding complications and lower warfarin dose requirements. Surprisingly, *CYP2C9* polymorphisms were only found to account for approximately 7–10% of the variation in warfarin dose [14], [18], [19].

Warfarin exerts its anti-coagulant effect by non-competitively inhibiting the action of vitamin K epoxide reductase complex subunit 1 (*VKORC1*) in an allosteric manner. *VKORC1* catalyses the conversion of vitamin K epoxide to reduced vitamin K, an essential co-factor for γ-glutamylcarboxylase (GGCX). GGCX is an enzyme which catalyses the γ-carboxylation of glutamic acid residues of clotting factors and proteins C, S and Z [20], [21]. Lately, functional genetic variants in the *VKORC1* gene have been found to affect the pharmacodynamics of warfarin and influence its dosage requirements in patients. Rieder et al., (2005) [22] have previously identified five haplotypes which are differentiated by five non-coding single nucleotide polymorphisms. These five haplotypes were found to segregate the patients into low- and high- dose groups and account for approximately 25% of the variability in warfarin doses. In a more recent study in Asian population, the *VKORC1* diplotypes were found to contribute to approximately 59.1% of the variability in warfarin dose requirement. In multivariate analysis, age, weight and genetic polymorphisms presenting *CYP2C9* and *VKORC1* accounted for 74.2% of the warfarin dose variability [23]. Approximately 25% of the variations in dose requirements still remained unexplained.

Although the availability of high-throughput genotyping capabilities can facilitate pharmacodynamics-based pharmacogenetic studies, pharmacoproteomic studies may provide additional information regarding variability in warfarin dose requirements in patients. Phenotypic traits are often the result of various proteins functioning in a concerted manner post-translationally and may be important in influencing interindividual variations to warfarin treatment [11]. The field of pharmacoproteomics may be more important than the pharmacogenetics of individual patients as it represents the effects of post-translational modifications of functional proteins which are responsible for the phenotypic effects and may serve as important biomarkers in patients. The objective of this exploratory study was to investigate the proteomic profile of patients receiving low- and high-dose warfarin and to perform correlative studies between genotypic and proteomic markers in the two groups of patients.

## Methods

### Patient's blood and tissue samples

The plasma proteomic profile of 53 patients (25 on low- and 28 on high-dose warfarin therapy) were analyzed in the present pilot study. These patients were part of a larger cohort of patients that participated in a previously reported study [24]. All human participants selected in this study have been approved by Singapore General Hospital Ethics Committee. Written informed consent has been obtained and all clinical investigations have been conducted according to the principles expressed in the Declaration of Helsinki. All patients were required to be on a stable maintenance dose of warfarin for at least one month with their international normalized ratio (INR) maintained between 2.0 and 3.0, and had no bleeding episodes. Patients with congestive heart failure (NYHA class 3 or greater), liver cirrhosis, thyroid disease or chronic gastrointestinal conditions were excluded from the study. Patients taking concurrent medications with potential to interact with warfarin included simvastatin (*n = *15) and omeprazole (*n = *18).

Healthy, non-cancerous liver tissues certified to be of malignancy free by pathologic examinations (n = 36) (from Chinese cancer patients undergoing hepatectomy for metastasis from a colonic primary malignancy) were available for the present genotyping and gene expression studies. All patients provided approved informed consent for participating in the study, and permission was also obtained from the institution's Ethics Committee.

### Pharmacogenetic analysis of *VKORC1* polymorphic variants

The pharmacogenetics of *VKORC1* [381T>C(rs7196161), 861C>A(rs17880887), 3673G>A(rs9923231), 5808T>G(rs2884737), 6484C>T(rs9934438), 6853C>G(rs8050984), 7566C>T(rs2359612) and 9041G>A(rs7294)] were determined in 53 patients receiving warfarin. The genotyping procedures were in accordance to recently published studies by our group [23], [24].

### Protein preparation and iTRAQ labeling

Protein from plasma samples was prepared in accordance to our previously published methodology [25]. Briefly, samples were centrifuged and the supernatant was stored. The protein levels were quantified using the 2-D Quant Kit (GE Healthcare). Each sample (100 µg) was precipitated by the addition of 4 volumes of cold acetone, dissolved in the solution buffer, denatured and cysteines blocked as described in the iTRAQ (Isobaric tags for relative and absolute quantitation) protocol (Applied Biosystems). All samples were analyzed in three independent analyses.

### 2-D Nano-LC-MS/MS analysis and data interpretation

In the first step of the separation, 3 µl of the combined peptide mixture were loaded onto the PolySulfoethyl A strong cation exchange column (SCX) (0.32×50 mm, 5 µm) [26]. The flow-through with peptides that do not bind to the SCX column is trapped in the Zorbax 300SB-C18 enrichment column and washed isocratically to remove excess reagent. In the second step, this enrichment column was switched into the solvent path of the nanopump and the peptides were eluted. An increasing concentration of acetonitrile elutes the concentrated sample and further separation was achieved onto the analytical Zorbax 300SB-C18 reversed-phase column. For electrospray analysis, the HP1200 LC system (Agilent Technologies) was interfaced to a QSTAR XL (Applied Biosystems-MDS Sciex) mass spectrometry. After the first analysis was completed, the enrichment column was switched again into the solvent path of the cation exchange column. Peptide identifications and quantization was performed using Proteinpilot software packages (Applied Biosystems). Each MS/MS spectrum was blasted against the human protein database and protein identifications were accepted based on Proteinpilot confidence scores, which are based on peptide scores, number of peptides and distance from nearest neighbor [26], [27]. Relative quantification of proteins in the case of iTRAQ was performed on the MS/MS scans and is the ratio of the areas under the peaks at 114, 115, 116, and 117 Da which represents the masses of the tags that correspond to the iTRAQ reagents.

### Total and free T3 and T4 immunoassay

The plasma samples of the patients (n = 53) were quantitated for the major thyroid hormones. Plasma levels of triiodothyronine (T_3_) and thyroxine (T_4_) (both total and free) were determined by chemiluminescent immunoassay using UniCel™ DxI 800 Access® Immunoassay System (Beckman Coulter, Fullerton, CA, USA) at the Department of Biochemistry, Singapore General Hospital as per the manufacturer's protocol. In brief, free triiodothyronine (FT_3_) and thyroxine (FT_4_) were determined by immunocapture and competitive-binding procedure while total triiodothyronine (TT_3_) and thyroxine (TT_4_) were measured in a one-step competitive-binding procedure. Reference intervals for the above assays were FT_3_ (3.2–5.3 pmol/L), FT_4_ (8.8–14.4 pmol/L), TT_3_ (1.1–2.6 nmol/L) and TT_4_ (74–144 nmol/L).

### RNA preparation and real-time RT-PCR

Total RNA was isolated from human liver samples (n = 36) using RNeasy Mini Kit (Qiagen). The amount and purity of the extracted total RNA was assessed in ND-1000 spectrophotometer by employing a module of extinction coefficient 40 to assess acceptable ratio of 260/280 ∼2.0 and 260/230 ratio range of 1.8–2.2. Prior to cDNA synthesis, total RNA (5 µg) from each sample was subjected to DNase treatment using TURBO DNA–*free™* (Ambion) to eliminate trace levels of genomic DNA contamination. After DNase treatment, 1 µg of total RNA was reverse-transcribed using high-capacity cDNA reverse transcription kits (Applied Biosystems) according to the manufacturer's procedure.

A 2 µL of cDNA sample was used as a template (i.e., 100 ng) to study the expression of *TTR* using TaqMan gene expression assay Hs00174914_m1 (Applied Biosystems). The reactions, in 20 µL, were performed in 7500 Fast Real-Time PCR systems (Applied Biosystems) as per the manufacturer's protocol. Constitutively expressed β–actin mRNA was simultaneously assayed by TaqMan human β–actin assay (part no.: 4333762F; Applied Biosystems) as an internal standard for sample normalization. All experiments were repeated three times, in triplicate. The expression of the *TTR* mRNA was calculated using the 2^-ΔC^T method as described by Livak and Schmittge (2001).

### HepG2 cell culture and warfarin exposure regimen

Human liver hepatoma cells (HepG2 cells) obtained from American Type Culture Collection (ATCC) were cultured in Dulbecco's modified Eagle's medium (DMEM), supplemented in 10% fetal bovine serum (FBS), 2 mM L-glutamine, 1 µM sodium pyruvate, and antibiotics penicillin (100 units/mL) and streptomycin (100 µg/mL). The cells seeded at ∼10^5^ cells/mL were maintained in 75-cm^2^ flasks at 37°C in a humidified 5% CO_2_ incubator with medium change every 3 days. Subcultures of cells were carried out from a 1∶4 split of 80–90% confluent monolayer using 0.125% Trypsin-EDTA solution. Under the same culture conditions, the HepG2 cells cultured in the DMEM without FBS are referred to as serum-minus HepG2 cells.

Warfarin stock prepared in methanol (95%) was used in culture medium to obtain the following final warfarin concentrations: 1.0 and 10 µg/ml (at <0.5% methanol). Twenty four hours before the cells were used in the experiments, the cells were sub-cultured at 2×10^6^ cells/mL in 25-cm^2^ flasks with fresh culture medium. At the start of the experiment, culture medium was removed and the monolayer was gently washed 2 times with pre-warmed (37°C) phosphate buffer saline (PBS, pH 7.3), and cells were then incubated in FBS-plus or FBS-minus DMEM containing warfarin to obtain the above final concentrations. The experimented cells (FBS-plus and FBS-minus) were harvested by scrapping out the cells at: 24 hrs (n = 3; flasks/concentration/time point). The scraped cells were transferred into 15 mL conical tubes by addition of 2 ml of cold PBS (5°C), and were pelleted immediately by centrifuging at 120× *g* for 10 min at 4°C. The harvested cell pellets were frozen immediately at −80°C for future experiments.

### Western immunoblotting

Total Protein from healthy hepatic tissues and warfarin exposed HepG2 cells were extracted using the Trizol method (Invitrogen) as per the manufacturer's protocol. Protein samples which are dissolved in 1% SDS were quantified using Bio-Rad *DC* Protein Assay. A measure of 200 µg protein is loaded per well to 12% SDS-PAGE gel. The proteins are transferred to Immobilon™ PVDF Transfer Membranes (0.45 µm) using Bio-Rad Criterion Cell. After transfer, membrane is incubated with 5% (w/v) non-fat dried milk (Bio-Rad) in PBS/T, which is used as the blocking buffer after transfer, for one hour at room temperature. After blocking, the membrane is incubated with Rabbit polyclonal Prealbumin antibody from abcam® (ab16006) which is diluted in PBS/T (1∶3000) at 4°C placed on a rocker. Next day, the membrane is washed with PBS/T for 10 minute each for three times and incubated with Goat Anti-Rabbit Ig HRP Conjugate (Sigma-Aldrich®) diluted in 5% non-fat dried milk PBS/T (1∶7500) at room temperature for one hour. Membrane is washed three times at 10 min interval with wash buffer (PBS/T). Bands are developed in CL-XPosure Film from pierce using Super signal West Pico Chemiluminescent Substrate. For endogenous loading control, we used Anti-GAPDH antibody produced in rabbit from Sigma-Aldrich® (G9545). TTR expression was then normalized against the endogenous control using the Multi Gauge Ver. 3.0 Software (FUJI FILM).

### OptEIA™ human IL-6 ELISA

IL-6 concentrations in plasma samples (low-dose warfarin, N = 39; high-dose warfarin, N = 37) were determined in duplicate by OptEIA™ ELISA (BD Biosciences, San Jose, U.S.A) in accordance with the manufacturer's protocol. The assay utilized a human monoclonal antibody specific to IL-6 and biotinylated anti-human IL-6 monoclonal antibody mixed with streptavidin-horseradish peroxidase conjugate for detection at 450 nm. The minimal detection sensitivity of the assay was 2.2 pg/mL and intra- and inter-assay coefficients of variation were 4.1 to 10.8, and 7.9 to 10.9, respectively. In order to acquire minimal baseline (2.2 pg/mL) for all the plasma samples, the initial additions of ELISA diluent (i.e., 50 µL/well) were spiked with the standard IL-6 to the concentration 3.0 pg/mL. An appropriate internal control was maintained in each assay to correct for the spiked base line for absolute IL-6 quantification. The absorbance of the IL-6 standards were fit by linear regression (R^2^ = 0.99) and the amount of IL-6 in the samples was quantified on the basis of this equation.

### Statistical analysis

The non-parametric Mann-Whitney U test and Kruskal-Wallis test were used to assess differences in *VKORC1* diplotype classes and warfarin dose levels association with different parameters TTR, thyroid hormones and IL-6. The level of significance was set as *P*<0.05 for all comparisons. All statistical analyses were performed using SPSS 16.0 (SPSS, Chicago, IL, USA).

## Results

### Patient demographics

Approximately 70% of the patients were males and the median age of the patients were 63 years (range: 46 to 75 years) and 50 years (range: 29 to 81 years) in the low- and high-dose warfarin groups, respectively. The median warfarin dose in patients receiving low-dose warfarin was 2.5-fold lower compared to patients receiving high-dose warfarin [low-dose versus high dose: 2.5 mg (range: 1 mg to 3.5 mg) vs 6.25 mg (range: 4.5 mg to 10.5 mg)]. Indications for warfarin therapy included deep vein thrombosis (44%) and cardiovascular conditions (atrial fibrillation, mitral valve regurgitation, transient ischaemic attack and stroke), (56%).

### Pharmacogenetics of *VKORC1*

The genotype frequencies of *VKORC1* polymorphic variants were in Hardy-Weinberg equilibrium. The distribution of *VKORC1* diplotypes frequencies is summarized in Table 1. The majority of the patients in the low dose warfarin group carried the *VKORC1* H1H1 genotype (N = 23; 92%) while H1H7 diplotype was the predominant diplotype in the high dose group (N = 14; 50%). *VKORC1* diplotype data were not available in two patients.

**Table 1:** The distribution of *VKORC1* diplotypes in patients receiving low-dose (N = 25) and high-dose (N = 28) warfarin.

VKORC1	H1H1	H1H7	H1H9	H7H7	H7H8H9	Missing	TOTAL
Low Dose	23	2	0	0	0	0	25
High Dose	1	14	2	6	3	2	28

### Proteins identified by iTRAQ-coupled 2D LC-MS/MS

**Figure 1:**
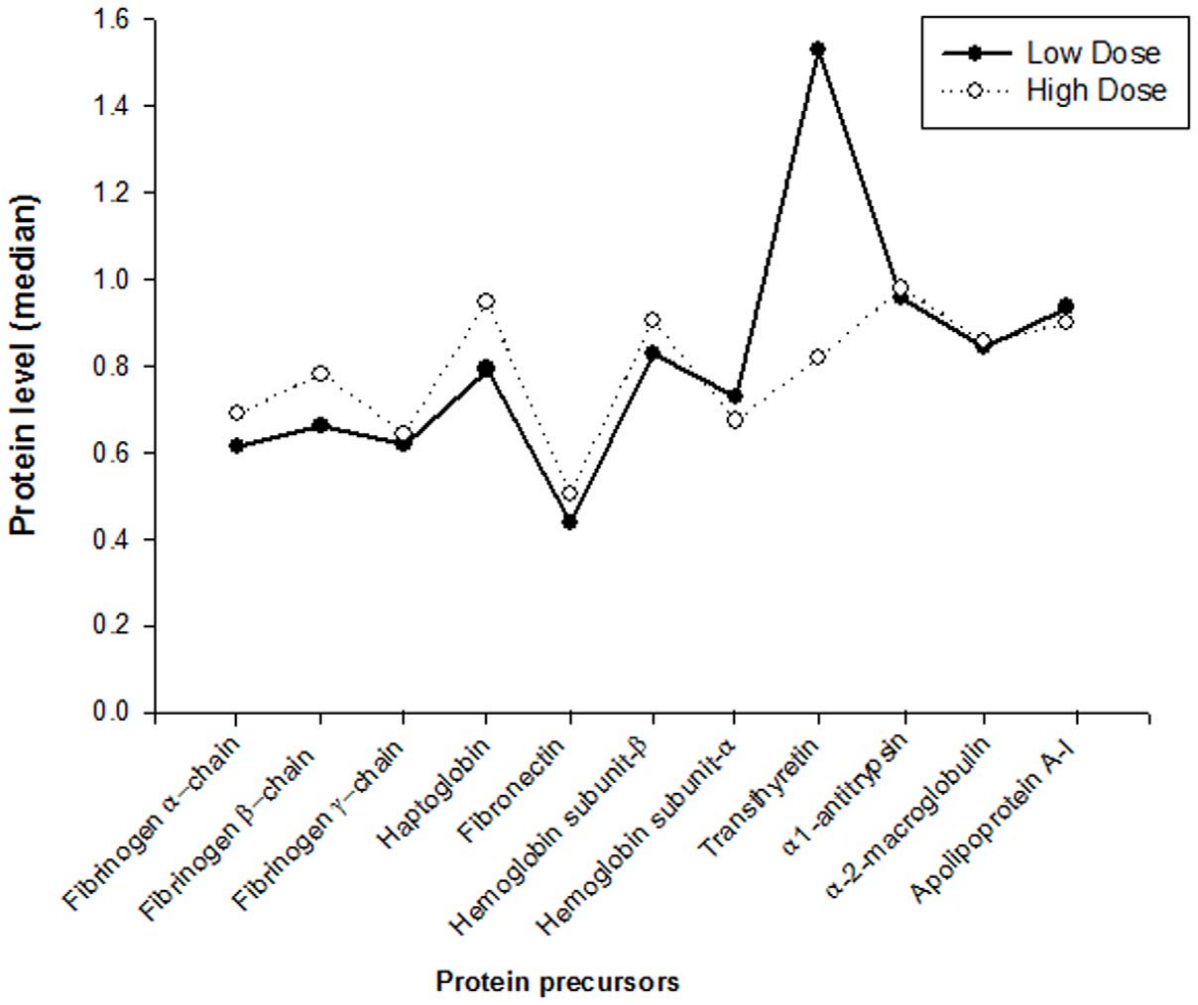
Differential expression of plasma protein levels in patients treated at low- and high-dose warfarin identified by iTRAQ-coupled LC-MS/MS analysis. A total of 163 proteins were identified and 11 proteins with significant expression levels were identified based on ProtScore with a cut-off value of 2.0 at 99% confidence value. Of the eleven proteins, the median expression level of transthyretin precursor was highly significant between patients receiving low- and high-dose warfarin group (low-dose: 1.53, range: 0.828 to 3.83; and high-dose: 0.818, range: 0.534 to 1.483; P<0.0001).

The proteomic profiles of patients receiving low- and high-dose warfarin therapy were investigated using iTRAQ-coupled 2D LC-MS/MS which was recently applied in the analysis of cellular protein profile in response to incubation with various chiral drugs, such as propranolol, ibuprofen and atenolol [25], [26], [27]. A total of 163 proteins were identified by iTRAQ-coupled LCMS/MS analysis. Identification of protein with significant expression level was based on the ProtScore with the cut-off at 2.0 and a confidence value of 99%. A total of 11 proteins satisfied these criteria and the results were reproducible when repeated in triplicates. As shown in Figure 1, of the differential proteins that were detected, the expression levels of TTR precursor was found to be significantly different between the patients requiring low- and high warfarin dose (P<0.0001).

### Influence of *VKORC1* diplotypes on *TTR* levels

**Figure 2:**
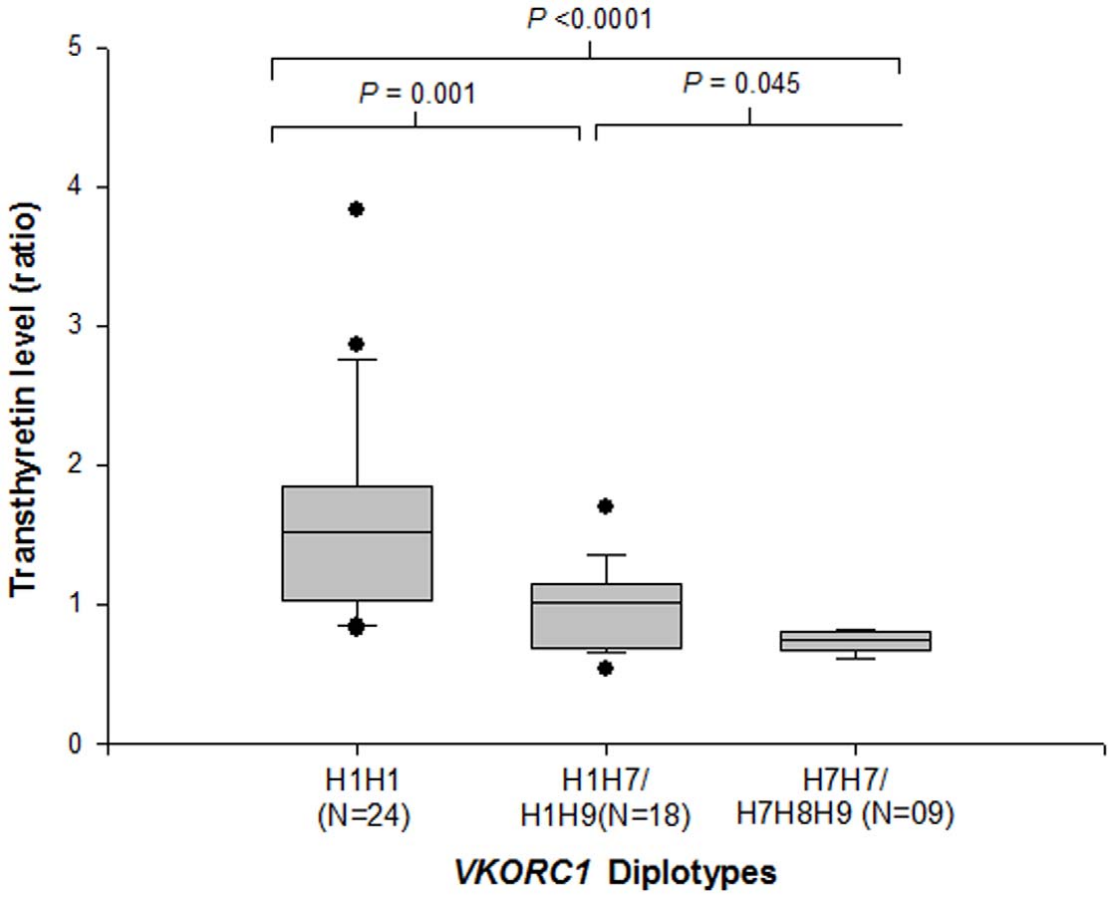
Influence of *VKORC1* diplotypes on TTR precursor level in warfarin treated Asian patients (N = 51). Patients carrying the H1H1 diplotype showed significantly higher levels of TTR precursor compared to patients harboring the H1H7/H1H9 (P = 0.001) and H7H7/H7H8H9 diplotypes (P<0.0001).

Figure 2 illustrates the influence of *VKORC1* diplotypes on transthyretin precursor levels. Patients harboring the H1H1 diplotype displayed significantly higher levels of the transthyretin precursor compared to patients harboring the H7H7 or H7/H8/H9 diplotypes, which were associated with high warfarin dose requirement (H1H1 vs. H7H7 vs. H7/H8/H9: values, P<0.0001).

### Influence of *VKORC1* diplotypes on thyroid T_3_ and T_4_ levels

**Figure 3:**
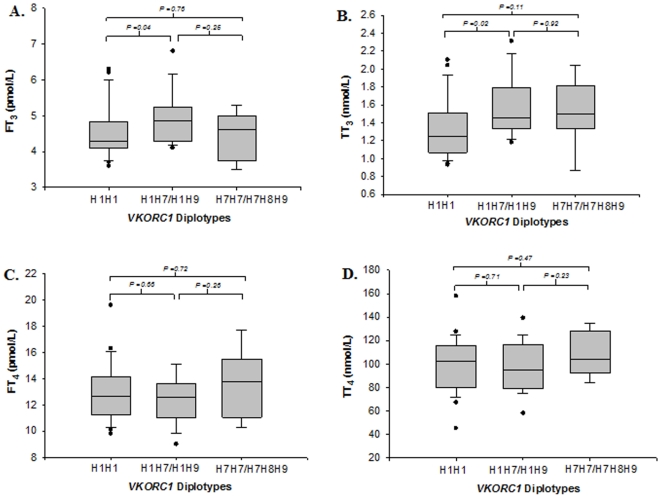
Effect of *VKORC1* diplotypes [H1H1 (n = 24), H1H7/H1H9 (n = 18) and H7H7/H7H8H9 (n = 9)] on (A) free T_3_ (FT_3_), (B) total T_3_ (TT_3_), (C) free T_4_ (FT_4_), and (D) total T_4_ (TT_4_) levels in patients receiving warfarin. Patients harboring the H1H1 diplotype group had significantly lower FT_3_ and TT_3_ levels compared with patients carrying the high dose (H1H7/H1H9) associated diplotype groups (Figures 3A and B; P<0.05 in each case). These findings probably reflect the higher levels of TTR expressed in patients carrying the low dose H1H1 diplotype, which probably leads to lower levels of FT_3_ as well as TT_3_.

**Figure 4:**
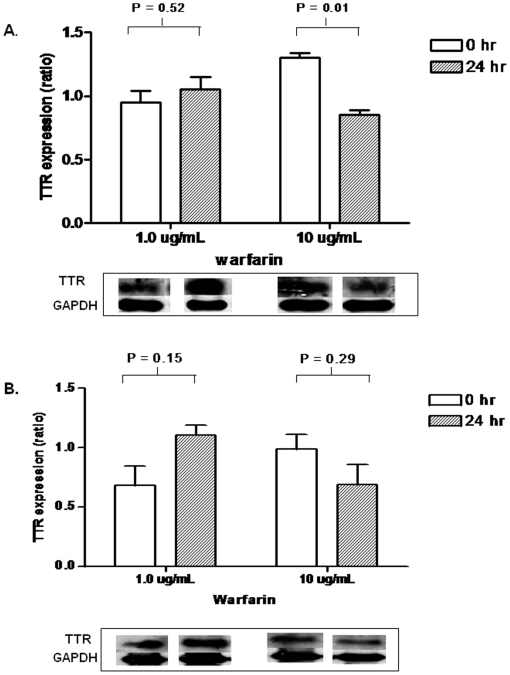
Immunoblot expressions of TTR levels in HepG2 cells exposed to low (1.0 µg/mL) and high (10 ug/mL) concentrations of warfarin for 24 hrs. TTR expression levels were determined in (A) serum-rich, and (B) serum-free conditions to exclude the possibility of false positive results. The data (mean ± S.E.) represents expression ratios of biological replicates normalized to GAPDH. The decrease in expression of TTR was significantly greater in HepG2 cells exposed to higher concentration (10 ug/mL) of warfarin under serum-rich conditions (Fig. 4A; P = 0.01).

The free and bound T_3_ and T_4_ levels were within the normal physiological range in all patients The median levels of FT_3_, FT_4_, TT_3_ and TT_4_ were 4.3 pmol/L (range: 3.6–6.3 pmol/L), 12.6 pmol/L (range: 9.8–19.6 pmol/L), 1.3 nmol/L (0.9–2.1 nmol/L) 101 nmol/L (range: 45–158 nmol/L) respectively in the low dose warfarin group (n-25) and 4.7 pmol/L (range: 2.5–6.8 pmol/L), 12.6 pmol/L (range: 9–17.7 pmol/L), 1.5 nmol/L (0.9–2.3 nmol/L) and 98 nmol/L (range: 58–139 nmol/L), respectively in the high dose warfarin group (n = 28). Figure 3A, B, C and D depicts the influence of *VKORC1* diplotypes on plasma FT_3_, FT_4_, TT_3_ and TT_4_ hormone levels. Significant differences were noted for FT_3_ and TT_3_ between patients harbouring H1H1 and H1H7/H1H9 diplotypes (Figure 4A and B, respectively) but not between patients carrying the high dose diplotypic groups (H1H7/H1H9 and H7H7/H7H8H9). The effect of *VKORC1* diplotypes on FT_4_ and TT_4_ were non-significant.

### *TTR* mRNA expression in hepatic tissues

The impact of *VKORC1* diplotypes, H1H1, H1H7 and H7H7, on the hepatic mRNA expression of *TTR* was investigated in 33 healthy hepatic tissue samples. The median *TTR* mRNA expression levels were shown to be similar (P = 0.88) across different *VKORC1* diplotypes (data not shown). The median (range) *TTR* mRNA expression level in hepatic tissues harboring H1H1 diplotype [259.16 (18.31–2691.12)] was comparable to that of hepatic tissues harboring H1H7/H7H7 diplotypes [273.71 (35.83–1023.66)]. We further evaluated the influence of *VKORC1* diplotypes on protein expression of TTR (data not shown). Similar to the *TTR* mRNA expression, the GAPDH normalized ratios of *TTR* protein expression levels were not found to be significantly different between hepatic tissues harboring different *VKORC1* diplotypes.

### *TTR* protein expression in HepG2 cells

In order to explain the negative correlation between the dose dependent warfarin treatment and TTR expression, we carried out an in vitro experiment using HepG2 cells which were exposed to low (1.0 µg/ml) and high (10 µg/ml) dose warfarin treatment for 24 hrs (with or without serum). Compared with control (0 hr), an average of 2.3 fold difference in the TTR protein expression was observed in cells exposed to low and high dose warfarin at 24 hrs (Figure 4A and B).

### IL-6 quantification of low- and high-dose warfarin patients

**Figure 5:**
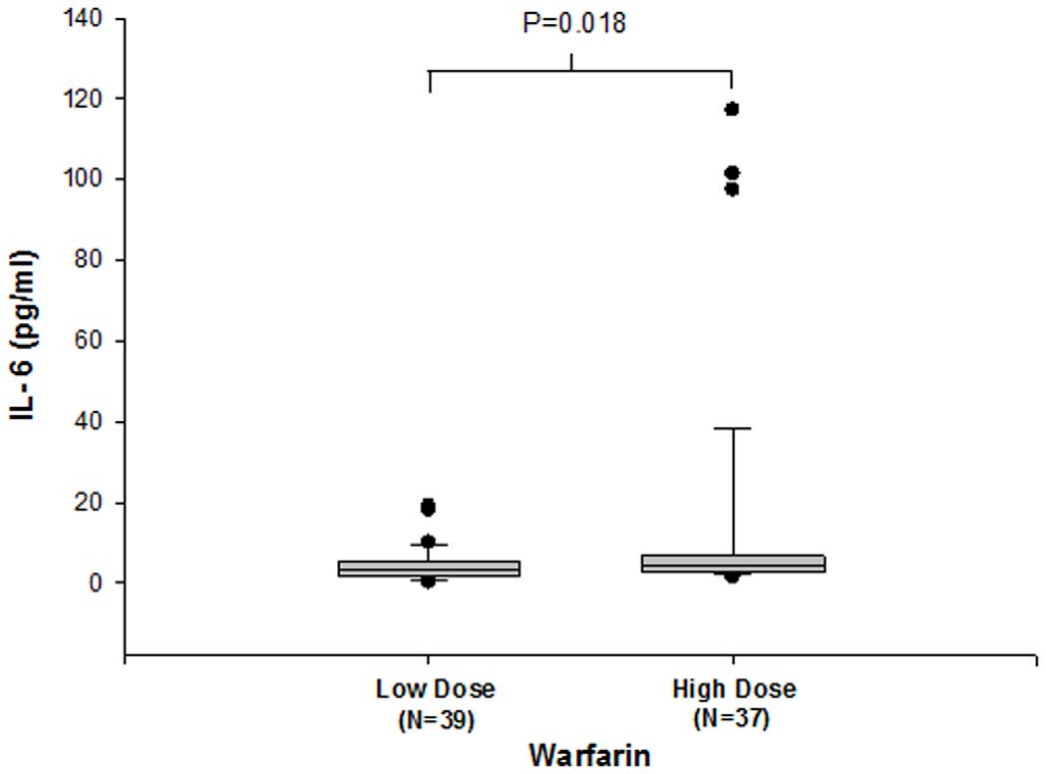
OptEIA™ quantification of IL-6 from low- and high-dose warfarin treated patients. Box plot shows the median IL-6 concentrations of low-dose (3.3 pg/mL; range: 0.14 to 19.05 pg/mL) and high-dose (4.4 pg/mL; range: 1.4 to 117.16 pg/mL) warfarin treated groups. Patients on high-dose warfarin (N = 37) showed significantly increased levels of IL-6 in comparison with those on low-dose (N = 39) warfarin [*P* = 0.018].

Interleukin-6 is an inflammatory cytokine and its secretion has been previously shown to vary according to warfarin levels[28], [29]. We thus sought to investigate the IL-6 levels in plasma samples of patients receiving low- and high-dose warfarin (Figure 5). The median IL-6 concentrations were 3.3 pg/mL (range: 0.14–19.05 pg/mL) and 4.4 pg/mL (range: 1.4–117.16 pg/mL), respectively in the low-dose and high-dose warfarin (*P* = 0.018).

## Discussion

The field of pharmacoproteomics is fast expanding and is proving to be a useful adjunct to pharmacogenetics and pharmacogenomics in drug development, diagnostics, drug safety and toxicology studies. In addition to pharmacogenetic factors, pharmacoproteomic profiling in patients has several associated advantages with regards to personalized drug therapy: firstly, it is directly associated with the observed phenotypic changes following drug therapy and secondly, it provides a better representation of the true nature of interpatient variations that are often observed between patients. The objective of the present exploratory study was to investigate for warfarin-specific biomarkers by performing collective genetic and proteomic profiling in patients who had received low- and high-dose warfarin.

Our study on plasma protein profiling showed that TTR precursor (54,980 Da.) was differentially expressed in patients who received low- and high-dose warfarin. Patients receiving low-dose warfarin were found to express higher TTR protein in plasma than patients in the high-dose group (Figure 1; *P*<0.0001). In addition, an inverse relationship was observed between *VKORC1* diplotype status and TTR levels in this exploratory study. Patients carrying the H1H1 diplotype displayed higher expression of TTR precursor whereas those carrying the H1H7/H1H9 diplotype showed lower expression of TTR precursor (Figure 2). We had previously established that *VKORC1* mRNA is differentially expressed in hepatic tissues, with lower levels in patients carrying the H1H1 diplotype and higher levels in patients carrying the H1H7 diplotype[30]. To investigate if a similar relationship was inherent between hepatic *VKORC1* and *TTR* mRNA expression, real-time PCR and protein immunoblot analysis were carried out. Both analyses revealed absence of any significant difference in *TTR* mRNA and protein levels between the different *VKORC1* genotypic groups.

TTR is predominantly produced in the liver and circulating TTR in plasma has approximately 100-fold lower affinity for thyroid hormones compared with thyroid binding globulin (TBG). Approximately 0.5% of circulating TTR is involved in the secondary transport of thyroid hormones T_3_ and T_4_ while a major portion exists in bound form to retinol binding protein[31], [32], [33]. A recent estimate revealed that most of the circulating TTR (0.15–0.3 mg/ml; 3–6 µM) is present in the unbound form and may possess multiple undiscovered biological functions[34]. Accordingly, changes in plasma TTR concentrations will be expected to have relatively little effect on the serum concentrations of thyroid hormones[33]. Although there were significant differences observed for FT_3_ and TT_3_ levels between patients carrying the low-dose (H1H1) and high-dose (H1H7/H1H9) associated diplotype groups (Figure 3A and B), the plasma levels of these hormones were within the normal biochemical range. These findings suggest that the influence of warfarin on plasma TTR levels is independent of the latter's negligible influence on circulating thyroid hormone levels and are therefore unlikely to affect physiological functions.

We next investigated in an *in vitro* model if the differential expression levels of TTR were due to warfarin treatment effect. HepG2 cells cultured under serum-minus as well as serum-plus conditions and exposed to low (1.0 µg/ml) and high (10 µg/ml) warfarin concentrations for 24 hrs showed the TTR expression patterns were inversely proportional to warfarin dosage (Figure 4A and B). Immunoblot analysis also revealed higher levels of TTR expression in cell culture system exposed to low concentrations of warfarin compared with those exposed to high concentrations of warfarin. To ensure that the observed effects were due to free warfarin concentrations, the experiments were conducted under serum-minus and serum-plus conditions. In a parallel study, warfarin showed negligible binding to TTR when studied using RP-HPLC as opposed to its being almost 100% bound with human albumin (data not shown). These findings suggest that warfarin may induce TTR expression in a dose-dependent manner. Further studies are required to validate these findings.

Increased IL-6 has been previously shown to decrease the transcription and translation of TTR in HepG2 cells[28], [35]. Also, low-dose warfarin has previously been reported to exert an anti-inflammatory effect through the suppression of IL-6 activity. In contrast, high-dose warfarin tends to activate pro-inflammatory signaling via increased IL-6 production[29], [36], [37]. In our study, concomitant with earlier findings, patients receiving high dose warfarin showed significantly increased levels of IL-6 in comparison with those receiving low-dose warfarin (Figure 5; p<0.05). The exact mechanism for this observation is not known. Kater et al[29] had previously reported similar findings and suggested that warfarin exerts its anti-inflammatory actions by affecting the phosphorylation of I-κB. Further studies are required to elucidate the mechanism underlying these observations.

In summary, this study showed that variations in TTR levels were associated with specific dosing regimens of warfarin. In addition to pharmacogenomic biomarkers currently being employed as an aid in the dosing of warfarin, pharmacoproteomic biomarkers such as TTR should also be tested in warfarin dosing algorithms. Future studies should investigate if the inclusion of both pharmacogenetic and pharmacoproteomic biomarkers may contribute to further improvement in variations of warfarin dose requirements.
